# Chemoenzymatic synthesis of macrocyclic peptides and polyketides via thioesterase-catalyzed macrocyclization

**DOI:** 10.3762/bjoc.20.66

**Published:** 2024-04-04

**Authors:** Senze Qiao, Zhongyu Cheng, Fuzhuo Li

**Affiliations:** 1 Department of Natural Medicine, School of Pharmacy, Fudan University, Shanghai, 201203, China; 2 Key Laboratory of Smart Drug Delivery (Ministry of Education), State Key Laboratory of Medical Neurobiology, Fudan University, Shanghai, 201203, China

**Keywords:** biosynthesis, chemoenzymatic synthesis, macrocyclic peptides, macrocyclic polyketides, thioesterase

## Abstract

Chemoenzymatic strategies that combine synthetic and enzymatic transformations offer efficient approaches to yield target molecules, which have been increasingly employed in the synthesis of bioactive natural products. In the biosynthesis of macrocyclic nonribosomal peptides, polyketides, and their hybrids, thioesterase (TE) domains play a significant role in late-stage macrocyclization. These domains can accept mimics of native substrates in vitro and exhibit potential for use in total synthesis. This review summarizes the recent advances of TE domains in the chemoenzymatic synthesis for these natural products that aim to address the common issues in classical synthetic approaches and increase synthetic efficiencies, which have the potential to facilitate further pharmaceutical research.

## Introduction

Nonribosomal peptides, polyketides, and their hybrids exhibit significant diversity and a broad spectrum of bioactivities [1–3]. Particularly, macrocycles from these three categories of natural products are vital resources for developing pharmaceuticals and drug candidates [4]. Many clinical pharmaceuticals with high market value, including the immunosuppressants cyclosporin and rapamycin, the antibiotics daptomycin and erythromycin, and the antitumor agent epothilone, all belong to these categories. Therefore, the rising demand for corresponding therapeutics requires effective and economical preparation methods [5]. In the synthesis of these natural products and their analogs, macrocyclization through linear precursors, the key step in the general routes, was typically accomplished via conventional chemical methodologies [6–7], keeps presenting an obstacle. Developing more efficient and diverse macrocyclization strategies is urgently needed to overcome issues such as insufficient regioselectivity, intermolecular oligomerization, the overuse of protective groups, and other drawbacks [8].

**Scheme 1:**
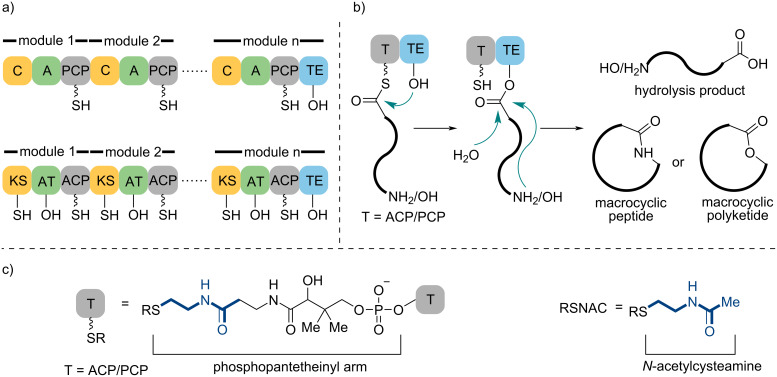
Brief introduction of thioesterase (TE) domain. (a) NRPS and PKS assembly lines. (b) Mechanism of TE domain-catalyzed macrocyclization and product release. (c) The common phosphopantetheinyl arm and its mimic (*N*-acetylcysteamine).

In the biosynthetic logic, these natural products are produced by the large and multifunctional enzymatic assembly, nonribosomal peptide synthases (NRPS), polyketide synthases (PKS), and hybrid NRPS/PKS systems, which are organized into sets of functional domains known as modules and function through a similar mechanism [9–12]. Each NRPS module is composed of three essential domains, namely adenylation (A), condensation (C), and peptidyl carrier protein (PCP). Each type I PKS module consists of three core domains containing acyltransferase (AT), ketosynthase (KS), and acyl carrier protein (ACP). PCP and ACP are collectively called thiolation domain (T). The sequence of monomers in the final product intimately correlates with the order of modules in the assembly line (Scheme 1a). Beyond several additional domains, including ketoreductase (KR), dehydratase (DH), enoyl reductase (ER), and methyltransferase (MT) domains and epimerase (E) domains, which are responsible for the structural diversity of natural products, both NRPS and PKS contain thioesterase (TE) domains in the final elongation module, which contribute to terminating biosynthesis [13–14]. Typically, TE domains cleave the thioester bond between the last PCP or ACP domain and the intermediate of polyketides or peptides, and form an ester bond. Then, they catalyze either intramolecular macrocyclization to give macrolactones or macrolactams with attacking of internal nucleophiles (alcohols or amine), or hydrolysis to release linear acids or peptides (Scheme 1b). Although TE domains may display cyclization and hydrolytic activities that are not easily predictable, related mechanism studies indicated that the pre-reaction states of the enzyme and substrate are critical for selectivity [15–16]. Thus, both the mutation of key residues in the active pocket and the addition of a nonionic detergent can increase the ratio of intramolecular nucleophilic attack, resulting in macrocyclic products via preorganization of substrate and enzyme in an active conformation [17–18].

Chemoenzymatic strategies, which merge practical enzymatic transformations with modern organic synthetic methods to increase the efficiency of synthetic approaches, have already shown a growing influence in the synthesis of bioactive natural products, pharmaceutical components, and other valuable molecules with the development of microbial genetics and enzyme engineering [19–22]. The comprehensive investigation of TE domains presents a significant opportunity to establish more efficient and environmentally friendly approaches toward bioactive macrocyclic peptides and macrolides [23]. Nevertheless, the native substrates of TE domains are tethered with PCP or ACP in biosynthetic pathways, which have to be simplified to chemically synthetic mimics before developing the enzymatic transformation. Due to *N*-acetylcysteamine (NAC) having a substructure to the phosphopantetheinyl arm of the carrier protein [24–25], the corresponding thioester can be recognized by TE domains and has become the most common substrate in enzymatic macrocyclizations (Scheme 1c). NAC thioester and other related mimics (such as coenzyme A (CoA), phosphopantetheine, and thiophenol) span the gap between the chemical synthesis and biosynthesis languages and expand the substrate promiscuity of TE domains. This bridge makes the in vitro TE-catalyzed macrocyclization possible and provides a potential to construct the analogs library of these bioactive macrocycles for further biological investigations.

This review presents representative examples of chemoenzymatic approaches for macrocyclic peptides, polyketides, and their hybrids employing TE domains, and particular attention is given to the strategies of mimics formation to demonstrate how biocatalysis provides an elegant link between chemistry and biology.

## Review

### Macrocyclic peptides

Since first being reported in the 1960s [26], solid-phase peptide synthesis (SPPS) has been an invaluable tool for preparing numerous peptides and even small proteins. In the chemoenzymatic synthesis of macrocyclic peptides, SPPS strategies provide highly efficient routes to access linear precursors, accelerating the development of enzymatic macrocyclization.

#### The tyrocidines

Tyrocidine A (**1**), a cyclic decapeptide isolated from *Bacillus brevis* [27], can penetrate the lipid phase of a Gram-positive inner cell membrane [28–29]. Despite exhibiting high antimicrobial activity, this compound also disrupts the membranes of higher mammalian cells, as evidenced by their pronounced hemolytic activity [30]. Establishing a concise and diverse method to produce analog libraries is critical for structure–activity relationship studies to enhance its specificity. Since the first total synthesis by Ohno and Izumiya in 1966 [31], tyrocidine A and its analogs have been synthesized by several groups employing viable strategies over the past half-century [32–35].

**Scheme 2:**
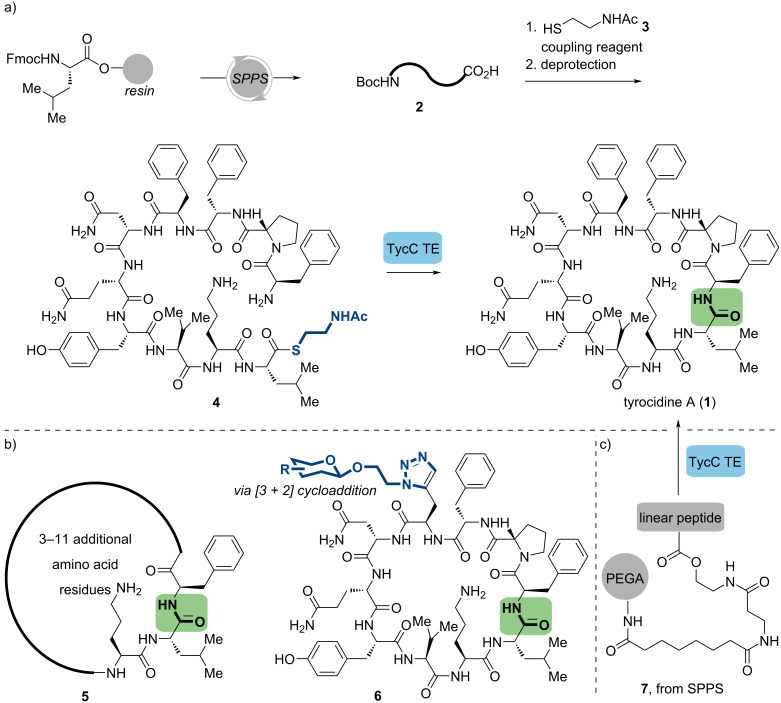
Chemoenzymatic synthesis of tyrocidine A and its analogs. (a) First-gen chemoenzymatic synthesis of tyrocidine A. (b) The analogs preparation catalyzed by TycC TE. (c) Second-gen chemoenzymatic synthesis of tyrocidine A.

Biosynthetically, the corresponding cluster consists of three NRPS, TycA-C, and at the C-terminus of TycC, the TE domain can catalyze a head-to-tail macrocyclization and deliver tyrocidines [30]. With a comprehensive understanding of its biosynthetic mechanism, Walsh and co-workers developed an elegant chemoenzymatic route in 2000 [36]. The synthesis commenced to construct linear decapeptide **2** by global SPPS method on 2-chlorotrityl resin. After coupling **2** with *N*-acetylcysteamine (NAC, **3**), the mimic of peptide-*S*-PCP, peptide-SNAC **4** was prepared. When incubated with purified TycC TE, the precursor was effectively converted into the macrocyclic tyrocidine A (**1**), exhibiting a low rate of substrate hydrolysis (Scheme 2a). In addition, TycC TE demonstrated a broad range of substrate tolerance, as it can cyclize a series of decapeptide-NACs that contain non-native residues in several positions and also form 6–14 residue cyclic peptides [37–38]. It should be noted that TycC TE was more sensitive to the amino acid changes near the site of ring closure. The alkyne-containing analogs were conjugated to a variety of azido sugars via copper(I)-catalyzed cycloaddition to obtain the corresponding carbohydrates modified tyrocidine derivatives [39], two of which exhibited a 6-fold better therapeutic index than the natural tyrocidine (Scheme 2b).

**Scheme 3:**
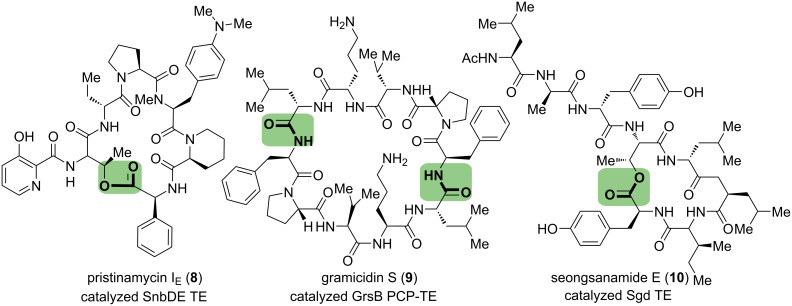
Representative examples of NAC-activated thioesters-mediated biocatalytic macrolactamization.

This seminal study illustrates that an isolated TE domain retains cyclization activity when peptide-SNAC is utilized to replace peptide-S-PCP. This strategy has been widely employed in the TE domains characterization and chemoenzymatic synthesis of other bioactive macrocyclic peptides, such as surfactin [40], streptogramin B [41], cereulide [42], seongsanamide E [43], etc. In 2005, Marahiel and co-workers accomplished the chemoenzymatic synthesis of type B streptogramin variants [41], including pristinamycin IE (**8**), which belongs to the class of depsipeptide antibiotics. Similarly, the linear peptide-SNAC was prepared through the SPPS method on 2-chlorotrityl resin, and macrolactonization was catalyzed by SnbDE TE, a thioesterase from pristinamycin I NRPS. This TE domain showed activity for hydroxy groups and amines to form either lactone or lactam, and the broad substrate scope made this strategy potent for modifying the bioactivity of streptogramin antibiotics. In 2007, the same laboratory identified the interactive TE domain of the gramicidin S synthetase GrsB [44]. Combined with the peptidyl carrier protein, GrsB PCP-TE was tested by using corresponding pentapeptides NAC thioester and thiophenol thioester, which led to the formation of the desired cyclic decapeptide lactam gramicidin S (**9**) through a sequential dimerization and cyclization process. Most recently, the synthesis of monocyclic depsipeptide, seongsanamide E (**10**), was reported by Boddy and co-workers via two different strategies [43]. On the one hand, the regular chemical approach, attempting Yonemitsu’s conditions to macrolactonize the seco-acid, was unsuccessful. On the other hand, the chemoenzymatic process using purified Sgd TE from its biosynthesis and a linear peptide SNAC substrate gave the macrocycle in an acceptable yield without epimerization (Scheme 3).

Although NAC-containing thioesters were widely employed, as described above, it had several limitations, such as possible Cα epimerization [45] during SNAC coupling and essential HPLC purification, which was generally difficult and time-consuming. Developing other different methods, exceptionally more straightforward approaches to access activated substrates, would solve this inevitable bottleneck and promote the utilization of TE domains as biocatalysts with tremendous potential. To this purpose, Walsh and co-workers developed the 2nd generation of tyrocidine chemoenzymatic approach utilizing a linear peptide immobilized on the solid-phase support poly(ethylene glycol) acrylate (PEGA) with a biomimetic linker to imitate peptide-S-PCP [46], which not only employed in the efficient cyclization of tyrocidine A (**1**) but also worked on hundreds of other linear substrates, some of which exhibited broad-spectrum activity against both Gram-positive and Gram-negative organisms. By combining natural-product biosynthesis and combinatorial solid-phase chemistry, this strategy has expanded the sequence space of macrocyclic peptides significantly (Scheme 2c).

#### The daptomycins

The calcium-dependent antibiotic (CDA, **11**), daptomycin (**12**), and A54145 are acidic lipopeptides isolated from *Streptomycetes*, which produce over 67% of naturally occurring antibiotics [47]. Notably, daptomycin, branded as Cubicin, was approved by the FDA as a last-resort antibiotic in 2003 for the treatment of infections caused by numerous Gram-positive bacterial strains [48], including methicillin-resistant *S. aureus* (MRSA) and vancomycin-resistant *S. aureus* (VRSA). However, the recent discovery of daptomycin-resistant *Enterococcus* and *S. aureus* provided the impetus to develop novel derivatives that enable more comprehensive structure–activity relationship (SAR) and resistance mechanism studies. Multiple approaches have been developed to address this challenge to produce daptomycin and its derivatives. These approaches include biosynthetic [49], chemoenzymatic [50], solid-phase [51], and solution-phase methods [52], but most only encompass modifications of the lipid chain and specific amino acid mutations.

**Scheme 4:**
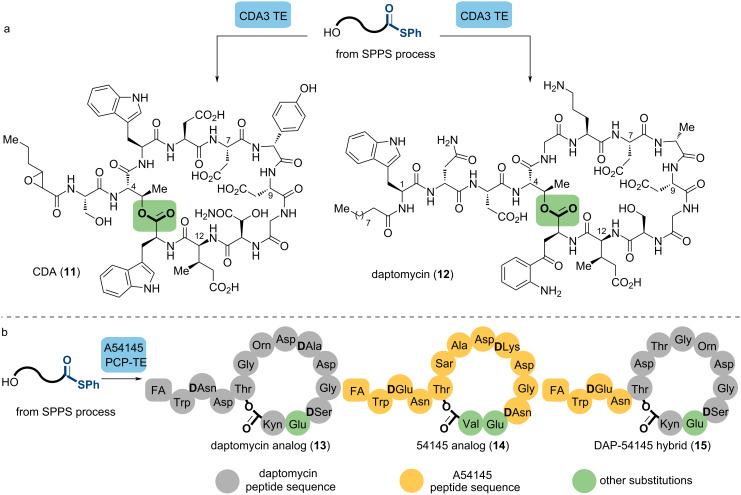
Chemoenzymatic synthesis of CDA, daptomycin and their analogs. (a) Biocatalytic macrocyclization of CDA and daptomycin mediated by thiophenol-activated esters. (b) Macrocyclization of daptomycin and A54145 catalyzed by A54145 PCP-TE.

Learning from the biosynthesis of these acidic lipopeptides, Marahiel and co-workers accomplished a chemoenzymatic synthesis of the calcium-dependent antibiotic (CDA,**11**) utilizing CDA3 TE, a cyclase derived from CDA synthetase [53]. To simulate the native peptide-S-PCP substrate, they evaluated four leaving groups: SNAC, coenzyme A (CoA), phosphopantetheine, and thiophenol. The thiophenol thioesters exhibited the highest cyclization rates, suggesting that chemical reactivity precedes cofactor recognition [54]. Moreover, CDA3 TE had a broad substrate spectrum, even indicating activity to cyclize daptomycin and its analogs, resulting in daptomycin (**12**) formation with a ratio of cyclization to hydrolysis of 3:1 (Scheme 4a). The significance of single amino acids for daptomycin bioactivity was evaluated, for instance, the substitution of ʟ-3-MeGlu_12_ by ʟ-Glu_12_ in Dap yielded a 7-fold increase of the MIC against *B. subtilis*.

However, this enzymatic macrocyclization can be limited by low yields due to competing hydrolysis or the fact that specific recognition elements intrinsic to cognate substrates can be needed for efficient substrate cyclization. To overcome this drawback, the Marahiel lab characterized two additional TE domains along with their associated peptidyl carrier proteins (PCPs): daptomycin and A54145 PCP-TE [55]. A series of thiophenol-activated precursors were tolerated by these enzymes to produce daptomycin derivatives, A54145 as well as hybrid molecules of the two compounds, which pushed forward the better understanding of the acidic lipopeptide structure–activity relationship (Scheme 4b).

#### Surugamide B

The cyclic octapeptides surugamides were isolated from several *Streptomyces* sp. and shown to be cathepsin B inhibitors [56–58]. According to a biosynthetic viewpoint, the corresponding modules consist of four sequential NRPS genes. However, none of them contain the thioesterase domain, which is essential for late-stage cyclization [59]. In 2018, Wakimoto, Kuranaga and co-workers reported the first total synthesis of surugamide B (**16**) in 34% overall yield through the general SPPS process and the macrocyclization at the biomimetic position (**17a**), which not only alleviated the epimerization in the macrolactamization process compared to other positions, but also enabled investigation of its biosynthetic pathway [60]. They also identified a stand-alone enzyme known as SurE, which is classified as a penicillin-binding protein (PBP) family and plays a role in chain termination and macrocyclization in the biosynthesis of surugamides. This PBP-type discrete TE was utilized in the chemoenzymatic synthesis of surugamide B with corresponding peptidyl-SNAC thioester (**17b**).

**Scheme 5:**
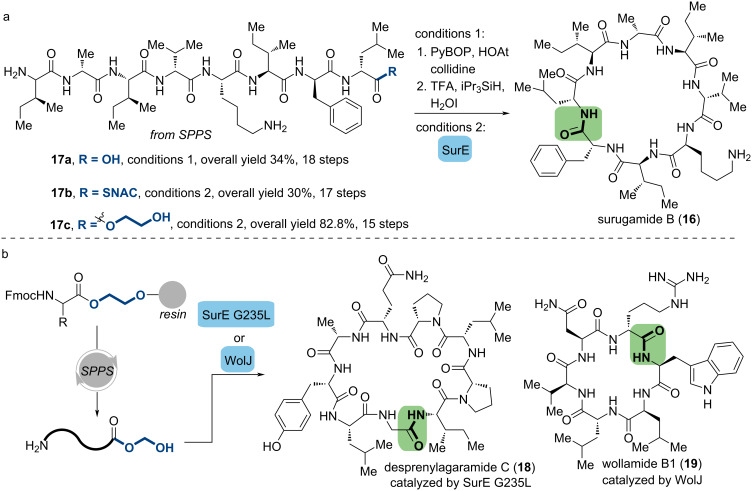
Chemoenzymatic synthesis of surugamide B and related natural products. (a) Three synthetic strategies of surugamide B. (b) Biocatalytic macrocyclization of desprenylagaramide C and wollamide B1 mediated by ethylene glycol-linked esters.

Most recently, it was observed that SurE exhibits a cyclization activity against a peptide methyl ester that is feeble but readily detectable [61]. This finding indicates that SurE has a high tolerance for leaving groups. In the light of this property, Wakimoto, Matsuda, and co-workers discovered that ethylene glycol (EG) can act as a linker on the resin before the SPPS, as well as a leaving group in further enzymatic cyclization (**17c**). Utilizing this approach, the overall yield of surugamide B (82.8%) was greater than the SNAC-based peptides with the same sequence used in the previous study (30%, Scheme 5a). In addition to investigating the high tolerance for different ring sizes, the sequential explorations of homologous wild-type enzymes and rational protein engineering have broadened the scope of the enzymatic macrolactamization [62]. Antibiotics, wollamide B1 (**18**) and desprenylagaramide (**19**), were prepared efficiently using the same manner catalyzed by homolog WolJ [63] and SurE G235L (Scheme 5b). Additionally, the above mentioned type I TE, TycC TE, can also tolerate ethylene glycol as a leaving group and gave tyrocidine A (**1**) in 70% yield, indicating that this convenient bifunctional linker may have a comparable applied range to *N*-acetylcysteamine.

Via bioinformatics analysis, Parkinson and co-workers most recently reported the characterization of Ulm16, the PBP-TE predicted to cyclize ulleungymycin. Compared with previously studied PBP-TEs, Ulm16 showed much higher efficiency and broader substrate scope, producing a variety of ullemgymycin-like hexapeptides and also working on the cyclization of penta- and tetrapeptides [64]. The findings illustrated that PBP-type discrete TEs would become potent tools for the construction of a noncanonical macrocyclic peptides library.

### Macrocyclic polyketides and PKS/NRPS hybrids

In contrast to the aforementioned NPRS macrocycles, the synthesis of macrocylic polyketides and PKS/NRPS hybrids is more challenging due to the absence of a streamlined preparation strategy such as solid-phase peptide synthesis (SPPS). In enzymology studies, it was common to hydrolyze the cyclic natural products in order to obtain the linear predecessors. Nevertheless, this method was unable to acquire substrate derivatives, restricting the exploration of the substrate scope for TE domains. Hence, it is necessary to produce linear precursors using concise stereoselective methodologies to facilitate the investigation of biocatalytic cyclization, a crucial feature for the chemoenzymatic synthesis of macrolides and PKS/NRPS hybrids.

#### The pikromycins

Methymycin (**20**) and pikromycin (**21**) are 12- and 14-membered macrolide antibiotics both isolated from *Streptomyces venezuelae* ATCC15439. The Kang lab reported the total synthesis of pikromycin and the aglycones in this family, 10-deoxymethynolide (**24**) and norbonolide (**25**), using asymmetric aldol reaction, Yamaguchi esterification, and ring-closing metathesis as key steps [65–66]. Nevertheless, the inherent complexity of these natural products demands high step counts, leading to low overall yield. According to the biosynthetic approach, these macrolides are produced by the type I PKS system, including thioesterase (TE)-catalyzed cyclization of the linear hexa- and heptaketide intermediates, post-PKS oxidation, and glycosylation [67]. Cane and co-workers reported that Pik TE, the TE domain found in pikromycin biosynthesis, has broad substrate tolerance for chain length variation, suggesting this enzyme can be a potent tool in the chemoenzymatic synthesis of macrolides [68].

**Scheme 6:**
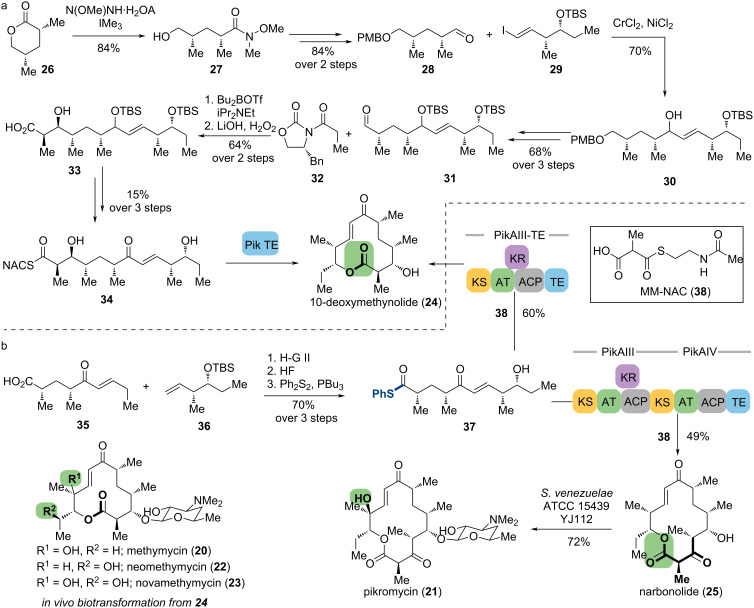
Chemoenzymatic synthesis of the pikromycins. (a) Macrocyclization of 10-deoxymethynolide catalyzed by Pik TE. (b) Biocatalytic synthesis of pikromycin, methymycin, and related natural products.

In 2005, Sherman and co-workers accomplished the total synthesis of 10-deoxymethynolide (**24**), via a late-stage TE-catalyzed marcolactonization [69]. The synthesis of linear peptide **34** commenced with the lactone opening of **26** to afford Weinreb amide **27**. Following primary alcohol protection and amide reduction, the aldehyde **28** was coupled with iodide **29** to afford **30** via Nozaki–Hiyama–Kishi coupling, which was then transformed into aldehyde **31** through several protecting group adjustments and the corresponding alcohol and Ley oxidation. After the preparation of **33** using Evans *syn*-aldol condensation as a critical step, **34** was produced by thioester formation, desilylation, and allylic oxidation. Incubating **34** with the purified Pik TE afforded 10-deoxymethynolide (**24**) as the exclusive product (Scheme 6a). Notably, using the corresponding C7-hydroxy NAC-hexaketide as substrate in this transformation resulted in exclusive hydrolysis to produce a seco-acid, indicating that Pik TE exhibits sensitivity to minor functional group changes of its natural substrates.

To increase the efficiency of pikromycins preparation, the Sherman lab developed a chemoenzymatic synthesis method through sequential propionate extension and marcocyclization catalyzed by fused PikAIII-TE and PikAIII-PikAIV modules. Based on this study, they established a preparative-scale approach toward the pikromycins family and their aglycones in 2013 [70]. The preparation of activated pentaketides (**37**) using asymmetric α-alkylation and cross metathesis as key reactions reduced the step counts from 14 to 11 steps. Replacing the extender unit from methylmalonyl-coenzyme A to its mimic MM-NAC (**38**) [68], and the substrate from NAC thioester to thiophenol-activated **37**, the PKS-mediated conversion proceeded with modest yield to 10-deoxymethynolide (60% yield) and acetylnarbonolide (49% yield) at preparative scale (>1 mmol), generating about 250 mg of both macrolactones. Using engineered variants of *S. venezuelae* ATCC 15439 designated strains DHS200141 [71] and YJ11242 [72], **24** and **25** were transformed to the corresponding macrolides through whole cell biotransformation to append ᴅ-desosamine and perform C–H oxidation(s) by the PikC monooxygenase (Scheme 6b). In contrast to Kang’s chemical synthesis route, this biotransformation provided a more efficient and productive strategy for the desoaminylation of macrolide aglycones. Combining in vitro and in vivo enzymatic reactions together, this chemoenzymatic platform exhibits the potential to access a broader range of unnatural macrolides with similar skeletons.

#### The juevnimicins

**Scheme 7:**
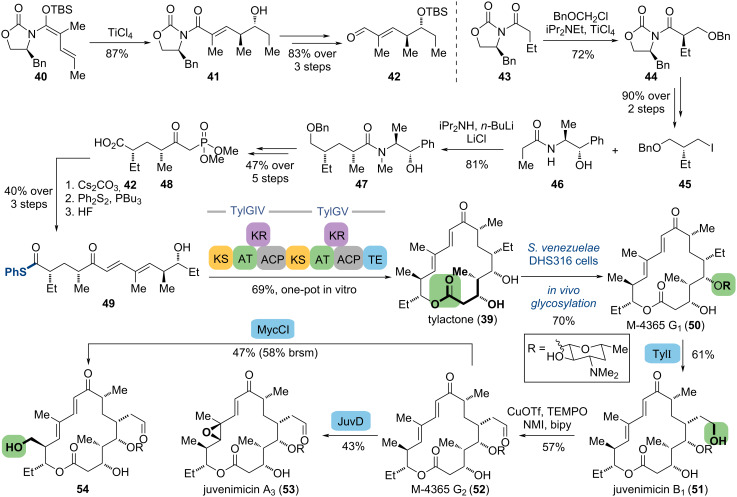
Chemoenzymatic synthesis of the juevnimicins.

Juvenimicins belong to a family of broad-spectrum macrolide antibiotics [73], playing an essential role in veterinary medicine, isolated from *Micromonospora chalcea* and *Micromonospora capillata*. They contain a 16-membered macrolide aglycone, tylactone (**39**), and a dimethylamino sugar, which are synthetically challenging. Therefore, the synthesis and evaluation of tylosin-related macrolides are hot topics in medicinal chemistry [66,74]. With the experience in pikromycins synthesis, Sherman and co-workers investigated the capabilities of two terminal polyketide synthases (PKSs) in juvenimicin biosynthesis in 2017 [75], which presented a chance to accomplish the chemoenzymatic total syntheses of tylactone and the juvenimicins (Scheme 7).

To generate an appropriately activated tylactone hexaketide intermediate **49**, two key fragments, aldehyde **42** and phosphonate **48**, were synthesized, employing Evans’ vinylogous aldol and Myers’ auxiliary-mediated alkylation reactions as key steps. By utilizing these two fragments, a Horner–Wadsworth–Emmons olefination, followed by thioester formation and desilylation, produced several different activated tylactone hexaketides, such as NAC thioester and thiophenol ester **49**, which serves as the precursor for downstream enzymatic assembly. Thiophenol ester **49** was accepted by the two terminal of tylosin PKS modules (TylGIV and TylGV) in vitro, which are responsible for the last four carbon atoms assembly and macrolactonization, leading to the formation of tylactone (**39**) in 69% yield. Furthermore, the *Streptomyces* strain *S. venezuelae* DHS316 [76] performed an in vivo glycosylation resulting in M-4365 G_1_ (**50**) in 15 linear steps and 4.6% overall yield from commercial resources. With regio- and stereoselective C–H hydroxylation and epoxidation using three P450s (TylI, JuvD and MycCI) involved in the biosynthesis of several different macrolides, eight additional macrolides were achieved from **50**, including juvenimicin B1, M-4365 G_2_, and juvenimicin A_3_. In the light of this approach, the following bioactive assay demonstrated that some of them exhibit comparable activities to the clinically approved antibiotics against Gram-positive strains while also enhancing activities against Gram-negative pathogens.

#### The cryptophycins

The cryptophycins are a large family of 16-membered ring depsipeptide natural products, which exhibit a potent ability to induce tubulin depolymerization [77], originally isolated from the cyanobacteria *Nostoc* sp. ATCC 53789 [78]. Notably, the cryptophycins cannot serve as substrates for *P*-glycoprotein and multiple drug resistance-associated proteins, making them attractive as chemotherapeutic options for treating vinca alkaloid- and taxol-resistant cancers [79]. Therefore, the pharmaceutical investigation of these natural products started for the first time when they were isolated in the early 1990s and has lasted until the present. A synthetic analog, cryptophycin 52, completed phase I clinical trials for the treatment of non-small-cell lung cancer and platinum-resistant ovarian cancer, but was halted in phase II due to dose-limiting peripheral neuropathy and limited efficacy in vivo [80]. However, this family of depsipeptides remains of therapeutic significance and has recently been explored as prospective payloads for antibody-drug conjugation [81–82].

**Scheme 8:**
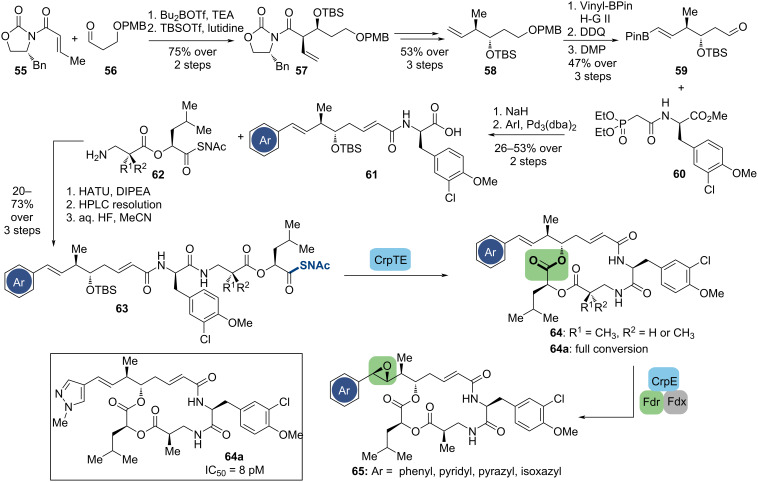
Chemoenzymatic synthesis of the cryptophycins.

Numerous synthetic approaches have been devised to deliver the cryptophytes skeleton, indicating that the most challenging steps are the regio- and stereospecific macrocyclization and epoxidation [83]. To address these problems, in 2005, Sherman and co-workers reported a chemoenzymatic approach through the stereospecific macrocyclization based on the identification of the thioesterase domain (CrpTE) from the cryptophycin biosynthetic pathway, which demonstrated that the CrpTE has both high efficiency in generating the 16-membered depsipeptide ring and broad tolerance for structural variation [84]. To simplify the synthetic process and expedite the comprehensive structure–activity relationship analysis, they modified the preparation of the linear chain elongation intermediate and conjugated the late-stage P450-catalyzed selective epoxidation with enzymatic macrocyclization in 2020 as shown in Scheme 8 [85].

According to their previous report [86], the production of fragments **61** was initiated by Evans’ asymmetric aldol and alcohol protection to generate **57**. Six-step route transformations, including cross metathesis, afforded aldehyde **59**, which was reacted with phosphonate **60** through Horner–Wadsworth–Emmons (HWE) olefination. Afterward, the coupling of **61** and **62**, followed by removing the silyl groups, gave the desired linear precursors **63**. The investigation of the enzymatic macrocyclization suggested that CrpTE is able to accept a diverse range of heteroaromatics. For instance, incorporation of a 4-methylpyrazole ring (**64a**) showed nearly complete conversion to product with no measurable starting substrate or hydrolytic byproducts. After producing these cryptophycin analogs utilizing the CrpTE, the selective epoxidation using wild-type CrpE P450 was examined with the assistance of the spinach reductase system, which provided a series of more complex analogs. Through biological evaluation, one of the most potent cryptophycin analogs (**64a**) to date has been identified, exhibiting significant potency against HCT-116 human colorectal cancer with an IC_50_ value of 8 pM [85].

## Conclusion

Macrocyclic peptides, polyketides, and their hybrids are natural products often used in different therapeutic areas. In the synthesis of these natural products and their analogs, more efficient macrocyclization strategies need to be developed to address the current issues, such as insufficient regioselectivity, intermolecular oligomerization, and the overuse of protective groups. The biosynthetic studies demonstrated that thioesterase (TE) domains exhibit a high level of chemoselectivity and regioselectivity in late-stage macrocyclizations. This review summarizes recent advances in combining thioesterase-catalyzed macrocyclization and typical chemical approaches in the rapid generation of these complex cyclic natural products and their analogs with exquisite biological activity. Moreover, multistep enzyme cascades simplify synthesis by reducing step counts, increasing yields, and minimizing waste generation as they couple different biotransformations in sequential reactions.

Although TE-mediated chemoenzymatic synthesis is becoming a prospective strategy, many challenges still need to be resolved, such as limited reaction solvents, enzyme stability, etc. Emerging research methods on bioinformatics, computational modeling, deep learning, protein engineering, and high-throughput screening will accelerate the pace of enzyme discovery to provide a broader platform of tools for employing chemoenzymatic strategies [64,87–89]. More chemoenzymatic approaches involving TE-catalyzed macrocyclization will keep expanding in scope and depth to explore previously inaccessible chemical space for discovering important therapeutically active natural product drug leads.

## Data Availability

Data sharing is not applicable as no new data was generated or analyzed in this study.
